# A Time-Series-Based New Behavior Trace Model for Crowd Workers That Ensures Quality Annotation

**DOI:** 10.3390/s21155007

**Published:** 2021-07-23

**Authors:** Fattoh Al-Qershi, Muhammad Al-Qurishi, Mehmet Sabih Aksoy, Mohammed Faisal, Mohammed Algabri

**Affiliations:** 1 Department of Information Systems, College of Computer and Information Sciences, King Saud University, Riyadh 11543, Saudi Arabia; msaksoy@ksu.edu.sa; 2 College of Computer and Information Sciences, King Saud University, Riyadh 11543, Saudi Arabia; qurishi@ksu.edu.sa; 3 College of Applied Computer Sciences, King Saud University, Riyadh 145111, Saudi Arabia; mfaisal@ksu.edu.sa; 4 Department of Computer Science, College of Computer and Information Sciences, King Saud University, Riyadh 11543, Saudi Arabia; malgabri@ksu.edu.sa

**Keywords:** annotation, crowdsourcing, classification, neural networks, quality control, time-series

## Abstract

Crowdsourcing is a new mode of value creation in which organizations leverage numerous Internet users to accomplish tasks. However, because these workers have different backgrounds and intentions, crowdsourcing suffers from quality concerns. In the literature, tracing the behavior of workers is preferred over other methodologies such as consensus methods and gold standard approaches. This paper proposes two novel models based on workers’ behavior for task classification. These models newly benefit from time-series features and characteristics. The first model uses multiple time-series features with a machine learning classifier. The second model converts time series into images using the recurrent characteristic and applies a convolutional neural network classifier. The proposed models surpass the current state of-the-art baselines in terms of performance. In terms of accuracy, our feature-based model achieved 83.8%, whereas our convolutional neural network model achieved 76.6%.

## 1. Introduction

Crowdsourcing, a concept first coined by Howe [1], refers to numerous people being involved in computing tasks to solve problems that are more difficult for computers than humans. It is an alternative mechanism for solving such problems at a lower cost. Therefore, many researchers have resorted to crowdsourcing as a preferable labeling choice in different domains, such as natural language processing [2] and image labeling [3]. Moreover, many researchers have incorporated crowdsourcing into studies on the COVID-19 pandemic [4], disasters [5], fake news [6], and preprocessing for deep learning applications [7,8]. Such studies leverage the availability of crowdsourcing platforms such as Amazon Mechanical Turk (AMT), as well as the abundant ordinary Internet users (i.e., crowd workers) [9]. Due to the heterogeneous nature of such workers, crowdsourcing is prone to quality concerns.

Crowd workers have different motivations, expertise, and backgrounds [10,11]. Moreover, human-specific factors (e.g., boredom, laziness, and inexperience), identity, and bias are other sources of quality errors [12]. Notably, numerous crowd workers are untrustworthy [13]. The percentage of spammers among crowd workers could be as high as 50% [14]. Moreover, crowd workers may attempt to maximize their monetary rewards by cheating using quick submission [15] or copy and pasting [16]. There are also sophisticated spammers who can evade certain anti-cheating crowdsourcing tests [17]. Others such as Sybil attackers can attack crowdsourcing tasks [17] using pseudo accounts to submit similar answers [18]. All of these problems can lead to deceptive results from such workers or high variability in quality due to variance in their effort or skills. In 2016 at least 20 million adults in the U.S. earned money by working from crowdsourcing like those found on Amazon Mechanical Turk (AMT), a number that is expected to rise with the growth of AI [19]. Furthermore, crowdsourced data processing is performed at scale at many tech companies, with tens of millions of dollars spent every year [20], so the quality improvement is a critical issue for those companies [21]. To attain high-quality results from crowdsourcing, various approaches have been proposed. A common technique is to evaluate crowd workers’ output using gold standards [22,23,24]. Unfortunately, many crowd workers may be rejected just due to bad luck on gold set questions. Moreover, spammers are aware of the use of such questions. Another widely used approach involves studying the relationship between crowd workers’ answers based on consensus methods such as majority voting [25,26,27,28]. One limitation of such a method is the high costs due to redundancy [29]. Moreover, such methods fail against collusion attacks by malicious workers [18]. Another approach uses motivations such as reputation in advanced quality measures [30].

An alternative way to cope with these problems is to study crowd workers’ behavior rather than their output. Compared with gold standard and consensus methods, behavior-based approaches can be generalized across tasks and do not only target closed questions. Moreover, these approaches are free from the cold-start problem, and they do not require workers’ historical annotation information.

Regrettably, only a few studies have targeted crowd workers’ behavior. Gadiraju et al. [16] studied different workers’ behavior limited to online surveys. Hirth et al. [31] examined different time aspects of worker behavior to find the most crucial features related to worker qualifications. Rzeszotarski and Kittur [32] estimated the labeling quality and accuracy of workers on different tasks based on task fingerprints and a set of statistical behavior features. Similarly, Kazai and Zitouni [29] collected experts’ behavior and trained a supervised classifier to detect poor crowd workers. However, none of these works have shared their source code or dataset. Leveraging from the open source of [33], Goyal et al. [34] are the only researchers who have shared their collected dataset. The present study benefited from this dataset and works.

The contributions of this paper can be summarized as follows:

- It proposes novel time-series-based models in the field of crowdsourcing quality control.
- It introduces two new models with various experiments. The first was based on time-series feature generation, showing the important features of crowd workers’ behavior. The other model was based on converting time series into heatmaps and then leveraging from their recurring characteristics to classify the tasks of crowd workers. The latter model establishes a baseline for research in the application of a lightweight deep learning model in the field of crowdsourcing workers’ assessment control.
- The proposed models possess superior performance. We demonstrated that our models outperform time-series state-of-the-art models such as dynamic time warping (DTW) and time-series support vector classifier (TS-SVC), as well as leading research works by Rzeszotarski and Kittur [32] and Goyal et al. [34].

The remainder of this paper is organized as follows. Section 2 presents the related work. Section 3 describes the methodology and the proposed models. Section 4 illustrates the experiment details and results as well as provides the discussion. Finally, Section 5 presents the conclusion and recommendations for future works.

## 2. Related Work

Generally, at least two types of approaches have been explored by researchers to obtain high-quality data from crowd workers, traditional and behavior-tracing approaches.

### 2.1. Traditional Approaches

In this line of research, quality control is applied to crowd workers’ responses. This can be based on other independent human evaluators, who are employed to assess the answers of other crowdsourcing workers [3]. Other studies have implemented consensus algorithms, where multiple answers are gathered for each task. These answers are then aggregated into the most likely answer or the so-called majority vote [35,36,37]. Unfortunately, this approach can greatly inflate costs due to the needed redundancy [38]. Furthermore, it is vulnerable to gaming, the majority effect, or at worst Sybil attacks [39]. Another traditional approach is to use gold standard data for filtering workers [22,23,24,40]. These gold data can be created by injecting a few ground truth labels from experts in rich crowd labeling [41] or by gathering a set of experts [22]. These standard data can also be generated automatically from just a few gold unit seeds [23]. A real-time system can evaluate crowd workers’ reliability using a collected reference set [24]. A shortcoming of such gold standard data is that they are not always available or applicable in some generative tasks, such as tagging and summarization. Both majority voting and gold data approaches assume that the response spaces are structured (i.e., the tasks have closed questions).

Other researchers have applied social motivations such as reputation as a pre-quality filtering approach. A social norm and welfare framework was simulated in [30] to mitigate the “free-riding” problem in crowdsourcing. Such an approach entails the drawback of evasion by malicious workers. Such workers can even acquire high reputation scores by accepting tasks that are unlikely to be rejected [32].

### 2.2. Behavior Tracing Approaches

A different line of research is focused in tracing the behavior of crowd workers when performing tasks. Kazai and Zitouni [29] stored experts’ behavior as behavior features (e.g., mouse movements, clicks, scrolls, and key-presses) of three different tasks. They applied it to training a gradient boosted decision tree classifier to detect poor crowd workers. Rzeszotarski and Kittur [32] proposed a task fingerprinting prototype that was mainly based on the recording of sequential logs of interface events. They collected statistical features related to time and browsing events, and then applied the prototype in different tasks and investigated decision trees to classify passing versus failing workers. Both of the aforementioned studies applied or necessitated expert behavioral data, which are difficult and costly to acquire in practice. In [31], the authors developed and applied the behavior approach of application layer monitoring. They studied three time aspects (i.e., completion, working phases, and consideration) using very fine-grained interaction level features to feed a support vector machine. One of their main findings was that a robust correlation existed between task interaction time and workers’ qualifications. They focused only on time features and neglected other behavior features. A visualization tool called CrowdScape was introduced in [42]. The researchers visualized both worker behavior and output information. They traced workers by analyzing and showing a highly granular user interface interaction level such as mouse movements, clicks, and focus changes. An analysis of search engine result pages was presented in [43], which studied the behavior of the assessors working on these pages. The authors defined different patterns based on time analysis and ratings accordingly and reported the possibilities of cheating and noncheating behaviors according to those patterns. Another work [16] was limited to an online survey, and collected data from two pre-defined workers. They presented five different groups of workers differentiated by their behavior. Restricted to a questionnaire, the researchers in [10] studied behavior in terms of five personality traits and observations of workers’ interactions with some task parameters, and defined another five types of workers.

A recent study [34] leveraged from [29,32] to generate richer behavior features investigated the feasibility of using these features of crowd workers for classifying the correctness of labels and predicting the labeling accuracy of workers. They applied a random forest classifier and a k-means clustering classifier for transforming sequence-like features. Next, they combined the random forest classifier with a co-training supervised naïve Bayes model for the sequence features. Lastly, some crowdsourcing quality research has focused on the consistency behavior of crowd workers, such [44,45]. Different from the aforementioned studies, the present study aimed to enrich the research by manipulating data as time-series data. This new angle of data manipulation will create opportunities for enhancing the performance of machine learning (ML) models, either by feeding ML models with spacious features as in our feature-based model or by leveraging deep learning models as in our second image-convolutional neural network (image-CNN) model.

## 3. Method and Materials

### 3.1. Dataset

To evaluate the performance of the proposed models against baselines and for experimental evaluation, we used the public dataset by Goyal et al. [34]. Its behavior data include 3984 HIT records. The data used for crowdsourcing tasks were collected from the 2014 NIST TREC Web Track.1. AMT workers were asked with judging the relevance of documents on 50 predefined topics. The judgment scale for those HITs was multiscale. For our experiments, we used a binary scale; that is, each record was either labeled as 1 if the worker correctly answered the task or 0 if he or she did not. The behavior features of these workers during their annotation were collected. The features were action-based, such as mouse movement, clicking, and scrolling, as well as time-based, such as total time and dwell time. We focused on action-based and converted them into time-series–based features as we will describe later.

### 3.2. Proposed Models

Human intelligent tasks (HITs) are tasks performed by crowd workers in crowdsourcing markets. For each HIT, the browsing behavior events of the worker are recorded. In our methodology, these browsing events were: e ∈ {mouse movement, key click, focus change, scroll, paste} [34].

In general, time series can be sampled regularly or irregularly through time and can therefore be represented as a vector of time stamps $t_{i}\;$, and associated measurements $x_{i}$. Let the sampling times be $t_{0},t_{1},\;\ldots ,t_{n}\;\mathrm{satisfying}\;0<t_{0}<\ldots <t_{n}$. If the time points are equally spaced, that is, $\left(t_{i+1}-t_{i}=\Delta \;\mathrm{for}\;\mathrm{all}\;i=0,\ldots ,n-1\;\mathrm{where}\;\Delta >0\;\mathrm{is}\;\mathrm{some}\;\mathrm{constant}\right)$ then the time series is regularly sampled; otherwise, it is an irregularly sampled time series.

In our case, the timestamps of the samples were not equally spaced, which meant that our samples were irregularly sampled time series. For each sample, the representation is an ordered vector of events $x=\left\{x_{1},x_{2},\ldots ,x_{n}\right\}$ with n measurements, where the associated measurement $x_{i}$ is the time at which the associated browsing action occurred. The HIT vectors have variable lengths depending on the number of actions a worker performed for each HIT. Finally, our two models were based on the manipulation of these samples as irregular time-series-like data. For example, consider a simple HIT log listing four browsing events over a 30-s period: ((t=0, mouse-move), (t=8, click), (t=19, focus-change), (t=30, click)). We recorded such an example as time-series data. This simple HIT is represented as vector ={0,8,19,30}; such vectors are the input samples for our two models, namely the feature-based model and the image-CNN model.

**Figure 1:**
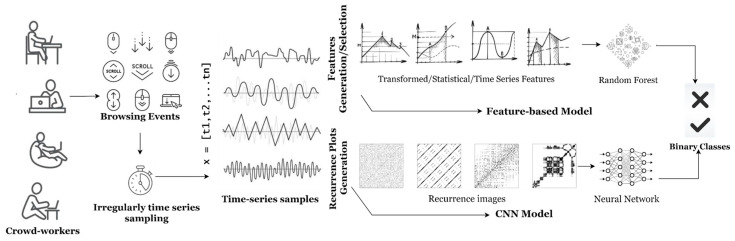
The proposed models.

Figure 1 depicts the two proposed models. First, the browsing behaviors of the crowd workers are captured as sets of events ∈e. Then, the timestamps of these events are stored for each HIT as time-series samples. These time-series are the inputs for both models. Whereas the feature-based model receives these samples as time-series features and uses an ML model, the image-CNN model receives them as sets of recurrence plot (RP) images/heatmaps. Finally, for both models, the output is a binary class that predicts whether the worker will label the HIT correctly or incorrectly.

#### 3.2.1. Feature-Based Model

In this model, we used feature generation and selection approaches to enhance the ML performance. Leveraging from transformed time-series samples, we generated a huge number of features to train ML models. Then, we selected and shed light on those features that remarkably enhanced the ML models’ performance and the most important features.

##### Feature Generation

For each time-series sample (workers’ HIT browsing behavior), we generated a set of global time-series features [46] (such as measures of its trend, entropy, and distribution). Then, we applied an ML classifier using these features to classify the samples (HITs). Global features of the time-series samples refer to algorithms that quantify patterns in time series across the full sample (rather than capturing subsequences). These global features are divided into different categories, which are described in the following four subsections.

- Statistical:

In this category, there are some simple statistical features, such as mean, median, variance, standard deviation, and sets of quantiles (where a quantile determines how many values in a distribution are above or below a certain limit). Others include count below the mean and ratio beyond sigma. Other more advanced features such as skewness and kurtosis are measures that define how far the distribution differs from a normal distribution. Another one is Benford correlation, which is a correlation resulting from the Newcomb-Benford’s Law distribution [47].

- Transformed:

This category contains two popular transformations. The first is the generation of Fourier transform coefficients. We generated the Fourier coefficients of one-dimensional discrete Fourier transform for real input using the fast Fourier transformation (FFT) algorithm as follows:

(1) $$A_{k}=\overset{n-1}{\underset{m=0}{\sum }}a_{m}e^{\left(-2\pi i\frac{mk}{n}\right)},\;k=0,\ldots ,n$$

where A is the returned coefficients and a is the time series. Furthermore, we leveraged FFT to extract statistical features of the absolute Fourier transform spectrum, such as spectral centroid (mean), variance, skew, and kurtosis. The second transformation is continuous wavelet transformation (CWT). We applied Mexican hat wavelet [48], which is the negative normalized second derivative of a Gaussian function:

(2) $$\psi \left(x\right)=\frac{2}{\pi^{\frac{1}{4}}\sqrt{3\sigma }}\left(\frac{x^{2}}{\sigma^{2}}-1\right)e^{\left(\frac{-x^{2}}{2\sigma^{2}}\right)}\;$$

where σ is the width parameter of the wavelet function  ψ.

- Information theory/entropy:

Different entropy measures were generated, such as sample and approximate entropy [49] and permutation entropy [50]. Other entropy measures were generated from the transformed one such as the binned entropy of the FFT.

- Time-series-related/others:

We also generated simple features related to the time-series, such as the sum, length, maximum, and minimum. Other specific time-series features were generated, including absolute energy, which is the sum over the squared values of the samples; energy ratio by chunks, which is the sum of squares or chunk i out of N chunks, and it is a ratio over the whole series; complexity estimation, which estimates how complex the time series is (e.g., whether it has more peaks or valleys); and symmetry, which checks whether the distribution of the sample is symmetrical.

##### Feature Selection/Reduction

Many features from the previous step have many sub-features. For example, there are nine quantiles and many coefficients for CWT and FFT, and many Fourier entropy bins. For each event e, there are approximately hundreds of generated features. As a result, the number of features returned from the generation step is huge. Therefore, we applied a feature selection/reduction approach to shed light on the most important features, and those remarkable features enhanced the ML models’ performance. We selected the extremely randomized trees classifier (ExtraTreeClassifier) for the feature selection/reduction. In our case, the HIT events in terms of time stamps were the vector samples. These samples were the input feature for this approach. The tsfresh [51] module was used for feature generation. In this step, the number of features generated was enormous. There were 3896 features, including the features in the section above, as well as others. These features were divided into training and testing sets for training a random forest (RF) model. Moreover, due to the huge number of features generated, we applied a feature selection approach to find the important features that mainly affect the ML model; then, we shed light on the most important features. We applied the ExtraTreesClassifier [52] for feature selection and then trained and tested the same RF model using these resulting features. We experimented with different thresholds for this step and investigated how they would affect the performance of the model. The list of 78 important features that returned from a mean threshold are shown in Table A1 at Appendix A.

#### 3.2.2. Image-CNN Model

In this model, we first converted the input time-series data into other behavior recurrent samples and then used the CNN model to train and test the new samples.

##### Recurrence Plot

Time series can be characterized by a featured recurrent behavior such as irregular periodicities. The recurrence plot (RP) is a famous tool for studying and visualizing such behaviors. It provides a graphical representation of the recurrent patterns in time-series signals. Eckmann et al. [53] proposed the RP as a matrix of pairwise recurrences of phase-space states. For a given trajectory $\vec{x_{i}}=\left(i=1,2,3,\ldots ,N\right)$ and $x_{i}\;\in \;R^{m}$, the RP is defined as follows:

(3) $$R_{i,j\;}=H\left(\varepsilon -\;||\vec{x_{i}}\;-\;\vec{x_{j}}||\;\right),\;i,\;j=1,\ldots ,N$$

where H(·) is the Heaviside function, ||·|| is the norm, and *ε* is a distance threshold. Basically, $R_{i,j}\equiv 1$ if time i recurs to a former (or later) state at j, and $R_{i,j}\equiv 0$ otherwise. In our case, we used a gray level instead of binarization to preserve much information (i.e., to which level $x_{j}$ is close to $x_{i}$)). The distance between two states $||\vec{x_{i}}-\vec{x_{j}}||\;$ was calculated using the Euclidean distance:

(4) $$D\left(x_{i},\;x_{j}\right)=\;\overset{m}{\underset{k=1}{\sum }}\sqrt{{\left(x_{ik}-x_{jk}\right)}^{2}})\;,m=2\;$$

Regarding ε, we ignored the distance threshold.

After calculating the distances, the recurrence matrices were stored as gray-level images (heatmaps) to be inputs for the model.

##### CNN Model

Compared with traditional ML methods, deep learning (DL) recently achieved great success in many computer science fields. CNNs are one of the most popular DL models. CNNs have achieved excellent performance in the image classification field [50]. We used a CNN to classify the images formed from the RP step as introduced in [51]. We used an enhancement of the model by concatenating the feature outputs from the CNN with auxiliary features to optimize the classifier. The details of CNN model are as follows.

The input to our network is an observation set $D=\left\{I_{i},y_{i};i=1,\ldots ,N\right\}$ containing *N* instances of $I_{i}\;\in \;{\mathbb{R}}^{d}$ (gray-scale image as the d-dimensional vector, *d* = 2 and number of channels = 1) with corresponding labels $y_{i}\;\in \;C$ (i.e., C≔{0,1} for binary classification) annotated by the workers. The goal is to learn the CNN model, represented by f:I→Y, from the labels that generalized well on unseen data:

(5) $$\hat{p}=f\left(I,y;\theta \right)$$

where $\hat{p}$ is the predicted label for an unseen image x, and θ is the learned model parameter.

The 2D convolution layer has the images I masked by a kernel K as follows:

(6) $$\left(K\;*\;I\right)\left(i,\;j\right)=\underset{m,n}{\sum }K\left(m,\;n\right)I\left(i+n,\;j+m\right)\;$$

where i,j are dimensions of the image and m,n are dimensions of the kernel.

Now, we consider a kernel K of size k×k, and x is a k×k patch of the image. The activation is obtained by sliding the k×k window and computing

(7) z(x)=φ(K ∗ x+b)

where b is a bias and φ is the activation function. The rectified linear unit (ReLU) [54] is used as follows:

(8) φ(x)=max(x,0) 

Then, a sub-sampling is used by adding a pooling layer (MaxPooling). After that, the flattening of the output is concatenated with a set of auxiliary features that are entered in two layers. These layers are the multilayer perceptron (or neural network). First, the fully connected hidden layer is followed by the output layer as follows.

For L=1 (hidden layer):

(9) $$a^{L}\left(x\right)=W^{L}h^{L-1}\left(x\right)+b^{L}$$

(10) $$h^{L}\left(x\right)=\delta \left(a^{L}\left(x\right)\right)\;$$

For L=L+1 (output layer):

(11) $$\;a^{L+1}\left(x\right)=W^{L+1}h^{L}\left(x\right)+b^{L+1}$$

(12) $$\;h^{L+1}\left(x\right)=\psi \left(a^{L+1}\left(x\right)\right)\;:=\hat{p}=f\left(I,\theta \right)$$

where $h^{0}\left(x\right)=z\left(x\right)$ that was calculated in (7); W is the weight vector $w_{g}=\left(w_{g,1},\ldots \;,w_{g,d}\right);$ and δ is the ReLU activation function for the fully connected layer. Finally, ψ is the activation function of the output layer, which is Sigmoid, and its output is the classification label p.

At each step, $W^{L}$ is a matrix for which the number of rows is the number of neurons (features x) in layer L and the number of columns is the number of neurons in layer +1. Regarding the architecture of our model, we defined the startup architecture and then we optimized it by tuning different hyper-parameters. The CNN model had one input channel of size 32 × 32, which represents the height and width of the gray images (the reason being that the time-series data had variable length, the longest sample being 128 and the smallest being 28; the average length was 32, so we selected the average length). These images were generated from RP mapping; 3985 samples were converted into gray images with variable dimensions. All were resized to the fixed 32 × 32 dimensions. The number of filters was 32; these filters had a 3 × 3 window size. Regarding the activation function, ReLU Equation (8) was implemented. The model had a subsampling step using the MaxPooling layer. To prevent the model from overfitting, a dropout with a 0.2 rate value was used. The model was flattened after that to enable the concatenation with auxiliary features. These auxiliary features were used to enhance our CNN model. They are shown in Table 1 (adapted from [32,34]). A fully connected layer with 64 neurons was used, followed by another dropout with a 0.5 rate. The last layer in the model is a single neuron with sigmoid activation to classify the images into two classes. For learning, we applied the Adam optimizer [55]. Binary cross entropy was used as the loss function. The number of epochs started with 200 and a batch size of 25. The validation ratio was 20% of the data.

For added details, we now describe the output dimensions of the layers. We have image I with dimensions of 32×32, one channel C (grayscale), and a filter size of 3×3. The default stride s of filter movement was 1. Furthermore, the convolution padding p was zero.

(13) The convolution output dimension=[((I−K)+2×p)/s]+1×C 

**Figure 2:**
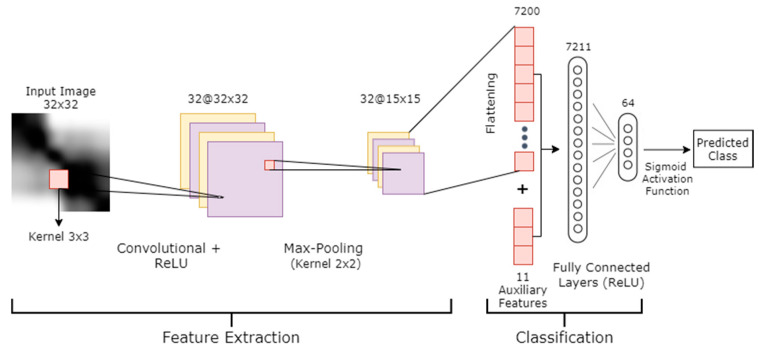
The image-CNN model architecture.

Therefore, the output dimension = 30×30×1. This was put into the pooling layer. In the MaxPooling2D layer in Keras, the default stride equals the pooling size, and our pooling size was 2×2; thus, s = 2. Using Equation (13) again, [((30−2)+2×0)/2]+1×1=15 . Consequently, the pooling output dimension = 15×15×1, and we had 32 filters, so the number of features was 15×15×32 = 7200. These features, concatenated with 11 auxiliary features, give the total number of features—7211. This means that $W^{L}$ in Equation (13) has dimensions of 7211×64, namely the features multiplied by the number of neurons of the fully connected layer. The last sigmoid layer produces a label of either 0 or 1. The detailed architecture of this model is presented in Figure 2.

## 4. Experimental Results and Evaluation

### 4.1. Experimental Results

#### 4.1.1. Evaluation Metrics

To evaluate the classification, we adopted accuracy as the comparison evaluation metric. Moreover, since the dataset had imbalance classes, we used the area under the curve (AUC) of the receiver operating characteristics (ROC) curve scores.

#### 4.1.2. Features-Based Model

- Parameters tuning:

One of the most common techniques for hyperparameters tuning in machine learning models is cross-validation (CV) using k number of folds. Random forests are not an exception. So, we first tuned the model hyperparameters using 10-fold CV to ensure that the model train and test 10 different data samples. We then calculated the average performance of these divided data samples. We further tuned the hyperparameters by randomizing search of optimized parameters. We defined a grid of hyperparameter ranges and random samples from the grid, performing 10-fold CV with each combination of values. We tuned different parameters using this method and the results were: no bootstrapping, no maximum depth, 131 features as maximum number, 7 minimum number of samples split, and 408 estimators. This optimization improved the performance for feature generation model by 1.7% in terms of accuracy and by non-noticeable improvement for AUC-ROC. Approximately, the feature selection model enhanced accuracy by 1% and it seems like the first model in terms of AUC-ROC.

- Feature generation and selection:

For the first model, 3896 features were generated. Therefore, we used ExtraTreesClassifier for feature selection and to demonstrate the important features from among the numerous features. Using ExtraTreesClassifier, we retrieved the importance of each feature. The importance represents the mean decrease in the Gini-impurity. Mean decrease in Gini is a famous measure of variable importance for estimating a target variable in decision trees, random forests, ExtraTrees models. The variable is the feature, and the target is the class. Different means of importance of all features are considered as importance thresholds. The higher the number, the more important the feature. Moreover, we used different thresholds to show how many important features were reduced by the model, as well as the accuracy and AUC-ROC score for each threshold. The thresholds are different means of the importance threshold that resulted from the ExtraTreesClassifier, as presented in Table 2. It reveals that both the accuracy and AUC-ROC fluctuated between 83% and 80% until the number of features reached 41, when they began to decrease to approximately 78%.

#### 4.1.3. Image-CNN Model

- Parameter tuning:

In this subsection, we outline the results of our CNN model using various hyper-parameters. Since the learning rate is one of the most important hyper-parameters to tune for training deep neural networks, we implemented an experiment to select the most suitable learning rate. We fixed the number of epochs to 300 and the dropout rate for the convolutional layer and dense layer to 0.5 and 0.25, respectively; consequently, the batch size was set to 50. Leveraging from [56], we set the learning rate range as base_lr = 0.1 and max_lr = $10^{-6}$. We started from a low learning rate and increased it exponentially for every batch using the step size, which we set to step_size = 20.

**Figure 3:**
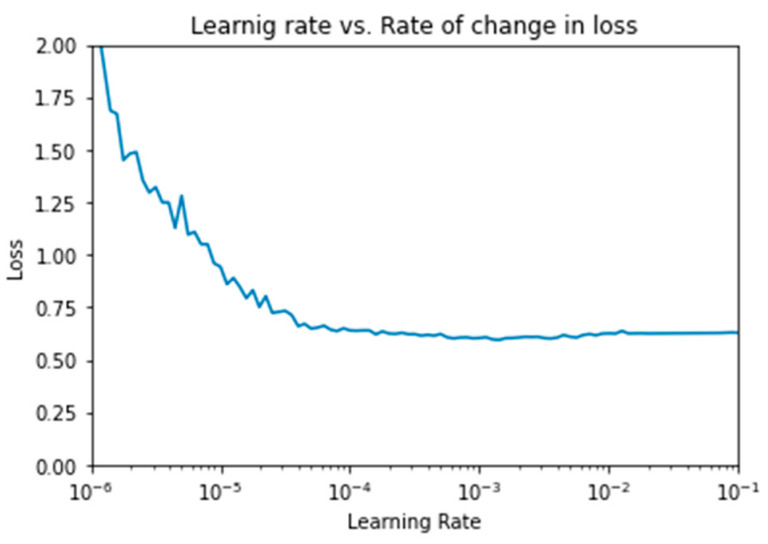
Optimized learning rate vs. changing in loss.

**Figure 4:**
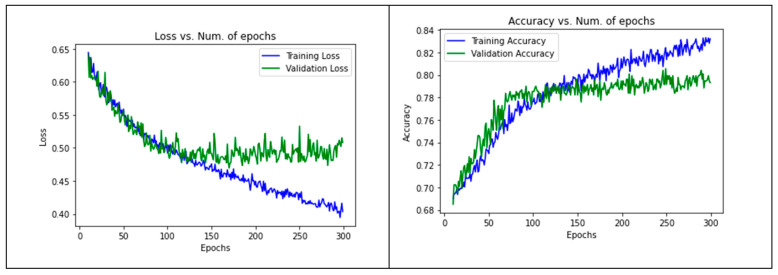
Optimized number of epochs.

As Figure 3 shows, there was a continuous decrease until reaching a stable state. The point of stability estimates the most suitable learning rate. We approximated it as $10^{-4}$. We fixed this learning rate for all subsequent hyper-parameters experiments. Regarding the number of epochs, we experimented with the training/validation loss and accuracy against 300 epochs, as shown in Figure 4. We found that at approximately 150 epochs, the validation accuracy and loss started to be fixed. This meant that the model started to be stable around 150 epochs and it seemed to enter in overfitting just after that. Therefore, we fixed the number of epochs to 150 for the following experiments.

Therefore, our initial hyper-parameters were as follows: learning rate:$\;10^{-4}$, epochs: 150, input dimensions 32 × 32, and dropout rates for the convolutional layer and dense layer = 0.5 and 0.25, respectively. Table 3 presents the use of different hyper-parameters and the corresponding training, validation accuracy, and loss. We selected the best validation accuracy and loss for fixing the hyper-parameters and then started to tune the next one.

**Figure 5:**
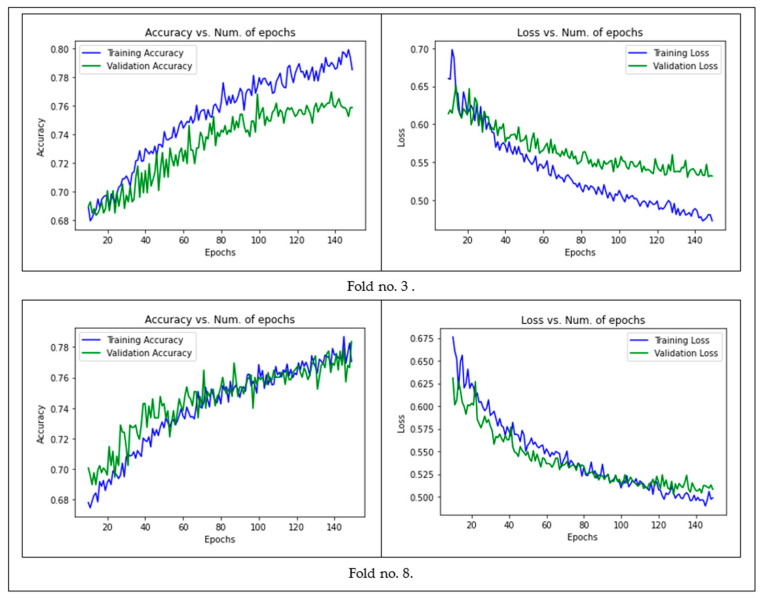
Training/validation accuracy and loss of the image-CNN model across the epochs.

First, we used batch sizes of 25, 50, and 75 and found that the optimal validation accuracy and loss were achieved with a batch size of 50. Second, for the convolutional layer, we used dropout rates of 0.25, 0.50, and 0.75. We noticed that the model attained the optimal validation accuracy and loss with the 0.25 dropout rate. The same was found with the fully connected layer where the 0.25 dropout rate achieved the best results. Finally, we tried some different dimensions for the images, namely 56 × 56, 28 × 28, and 32 × 32, as recommended in [57]. Regarding the training, validation loss, and accuracy across the epochs, since we conducted a k-folding experiment with k = 10, we only present two folds (the third and eighth epochs) in Figure 5.

#### 4.1.4. Baselines

The proposed models’ performances were compared with different baselines using the dataset of Goyal et al. [34]. The first baseline was a decision tree with aggregate features (DT-AF) classifier, which is a classifier model from Rzeszotarski and Kittur [32]. It uses behavior features in addition to updated aggregated behavior features of Goyal et al. [34]. The second baseline was a random forest [58] with the same aggregate features (RF-AF) classifier, which was a generalization of the DT-AF classifier. We applied some optimization for these two baselines like the parameters tuning in Section 4.1.2. Same parameters for RF-AF with our feature-based models, which are random forest algorithms. However, we tuned the parameters less with DT-AF since decision trees had fewer parameters to be tuned. In spite of that, DT-AF had noticeable enhancement compared to no improvement for RF-AF.

The third and fourth baselines were popular classifiers in time-series field. The third was k-nearest neighbor classifier using dynamic time warping (DTW) as a distance measure. This classifier has received enduring interest and been shown to be effective for time-series classification [59]. DWT is a popular comparison algorithm in time-series analysis that finds an optimal alignment between two given (time-dependent) sequences under certain restrictions [60], rather than measuring similarity or dis-similarity between two input times series using Euclidian distance between the corresponding points of the inputs. DTW is a very robust method to compare them using a sliding window instead of pair comparison. This is to ignore any phase shifts and speed between the inputs. We consider this simple approach, since it often produces better results than more complex classifiers [61].

The fourth and final baseline was a support vector classifier SVC for the time series data (TS-SVC). Support vector classifier (SVC) is an algorithm that searches for the optimal separating surface between classes using hyperplane. When there is a non-linearity relation between the data, such as in our case, SVC needs to apply a suitable kernel. In the domains that frequently use time-series data such as bioinformatics, there is increasing domain-kernels usage [62]. We applied SVC with such time-series called global alignment kernel [63]. This kernel implements a maximum smoothed DTW score across all possible alignments between the two compared time-series samples. TS-SVCs are promising methods for predicting different time-series domains such as financial [64] or biomedicine [65]. No dedicated optimization is applied for these models. We tried some parameter tuning like the number of neighbors, but no noticeable enhancement occurred. This, unfortunately, came with a large increase in time complexity.

#### 4.1.5. Parameters and Software

For the DT-AF baseline, we used the default settings. For the RF-AF model, we used an ensemble of 100 trees in the forest. We used the scikit-learn module [66] for these baselines. For DTW, we used two neighbors for distance calculations. For both the DTW and TS-SVC baselines, we implemented the corresponding Tslearn [67] libraries. For the division of data, we used 10-fold cross-validation and the test size was 20% for all models. The experiments were conducted on a machine with the following criteria: a GeForce GTX 980 GPU with 47 GB RAM, using Python 3.8 and Keras API in the Ubuntu 20.04 operating system.4.2.6. Comparison with baselines:

In this subsection, we present the classification metrics and results of the proposed methods and baselines. Our models achieved optimal accuracy. The two feature-based models attained accuracies of 83.8% and 81.8%, followed by the image-CNN model, which attained 76.6%. Furthermore, in terms of AUC-ROC, the two feature-based models won, achieving accuracies of 82% and 80.9%, followed by the image-CNN model, which achieved 72.3%. We believe that the image-CNN model was able to achieve better results; however, the number of samples was small, and such CNN deep networks require a huge amount of data. For DTW, we only used two neighbors since any increase in neighbors would increase the time complexity of the model without any noticeable enhancement in performance. TS-SVC had the same time complexity challenge, and the same observations were noted; specifically, any increase in the comparison numbers led to increased quadratic time complexity without a remarkable improvement in the results. Table 4 presents the results of the performance comparison against baselines in terms of accuracy and AUC-ROC with an average 10-fold cross-validation.

## 5. General Discussion

### 5.1. Feature-Based Models

The feature-based models achieved superior performance over analogous models such as [32,34]. A significant factor is the large number of features generated from our time-series transformation. Rather than having around twenty features in the baselines, our model has around 4k features in the first feature-based model and 41 features for the worst performance of the second model. The results showed that the feature-based models classified the workers well when their browsing events in HITs were captured as time-series samples. This gives the indication about the good consideration of crowd behavior as a time-series representation. Regarding the optimization of these models, we found that cross validation with main hyperparameter tuning raised the performance around 2%. This is a fine percentage. However, we did not expect more noticeable improvement for further optimization. These models have limited options in the optimizations. Regarding the most important features affecting the performance, as Appendix A shows, the majority were related to either mouse movement or focus change events. Mouse movement was the most significant event followed by focus change and then key clicks. In addition, scrolling and paste events seemed not to exhibit any noticeable enhancement in the ML models. This seems reasonable, since the workers in the HIT used more mouse movements than other events. The focus change gives a good indication about the intention of the workers to do a good work. Generally, among 78 features, we found that 41 features were related to mouse movement, followed by 20 related to focus change and only 16 related to key clicks. In particular, significant features were the transformed ones such as the CWT and FFT coefficients for both focus change and mouse movement. In addition, large sets of quantiles had significant impacts in the ML models. Other statistics such as mouse movement and focus change maximum, minimum, and mean were also important. This indicated that the statistical features of mouse movement and focus change remarkably affected the classifier performance. Mouse movement absolute energy and energy ratio by chunks were other important features. Moreover, especially for mouse movement, approximate entropy was returned as an important feature. This could be reasonable since approximate entropy is designed to work for small data samples (*n* < 50 points) [68], and we had *n* = 32.

### 5.2. Image-CNN Model

Differently, we selected a new model in this research scope compared to saturated ones such as random forest, decision trees [29,32,34]. Our image-CNN model archived comparable performance results despite the small number of behavior traces. Such shortage in data samples does not yield competitive performance using such neural networks models [34]. Therefore, one of the factors of such performance is the transformation of the numeric data into heatmap images. CNN is the most widely used deep learning model in the areas of image processing [69]. Many researchers used variations of CNNs for image classification and achieved superior performance such as [70,71,72]. Another factor is the intensive optimization for this model. We deepened the optimization in this model to enhance the performance because such models are rare/nonexistent in the research of classifying crowdsourcing workers using their behavior. This model presents a prospective beginning for further research on neural models such as deep CNN and long short-term memory (LSTM) models. For the optimization we implemented hyperparameter optimization HPO [73]. It consists in fixing the various hyperparameters of the model. We optimized global hyperparameters like learning rate, epochs, and regularization parameters such as dropout rates. We started the tuning from the learning rate since it is one of the most important factors [56]. Then, we gradually tuned other significance hyperparameter like number of epochs, batch size, dropout rate, and dimensions. The accuracy and loss monitoring are the key for such tuning. In terms of the number of epochs and the dropout rate, overfitting was alarming. We selected 150 epochs and dropout rates as 0.25 in both layers since other values for epochs and dropout rates led to overfitting. Regarding the image dimension, as expected, 32 × 32 provided the best results since it was equal to our average time-series sample length, which is 32.

### 5.3. Baselines

The baselines are of two types: (a) state-of-the-art models such as dynamic time warping (DTW) and time-series support vector classifier (TS-SVC). (b) Leading research works by Rzeszotarski and Kittur [32] and Goyal et al. [34]. Regarding their optimizations, for both DT-AF and RF-AF, in terms of accuracy, only DT-AF model achieved considerable enhancement. It reached the performance adjacent to RF-AF model. This could be interpreted based on the small number of features. A small number of features did not result in any performance disparity between decision trees and random forests. However, decision trees needed some more optimization. RF-AF model did not achieve any noticeable enhancement. In terms of AUC-ROC, there was no enhancement for either model. Even after optimizations, DT-AF and RF-AF models still achieved lower performance compared to the proposed models. Alternatively, we did not make any considerable optimizations for DTW or TS-SVC models. For DTW, we only used two neighbors since any increase in neighbors would increase the time complexity of the model without any noticeable enhancement in performance. TS-SVC had the same time complexity challenge, and the same observations were noted; specifically, any increase in the comparison numbers led to increased quadratic time complexity without a remarkable improvement in the results.

## 6. Conclusions

In this study, two new models were proposed to deal with the quality problems related to crowdsourcing. These models depended on time-series data. These data represented the browsing behavior of crowd workers. Each model dealt with the data differently. The feature-based model generated a huge number of features that fed an ML classifier. The richness of the features enhanced the classifier’s performance. Our experiments shed light on which features were the most important, and, consequently, on the remarkable browsing events that determine crowdsourced work quality. The image-CNN model gathered the time-series data as recurrent heatmaps and fed a CNN model. The two models provided a classification for HITs that predicted whether a HIT would be performed correctly by a worker based on his or her browsing behavior. Both models significantly outperformed the state-of-the-art and leading classifiers.

There are some limitations in our work. The training data are limited in this study regarding models such as image-CNN model. The performance of new promising AI approaches, such as deep learning, is strongly correlated with the amount of training data available. Therefore, further research is needed to exploit larger training data. This could be carried out either by creating an extensive dataset and then using this dataset as time series with recent models such as LSTM, or, alternatively, by implementing data augmentation for the generated recurrent images in this study. Undoubtedly, this will enhance the CNN performance, specially with extending the CNN model into deep CNN with more deep layers and more hyperparameter tuning. Another limitation is the transformation into irregular time series samples. Although the performance of the proposed models is noteworthy, having a likely regular time series sample will enhance the models remarkably. Therefore, in future work, we plan to resample time-series using different techniques. Rather than primitive methods such as shifting and imputing, we will exploit more advanced resampling approaches such as periodic identification [74] and causality analysis [75]. One more limitation is the performance of DTW and TS-SVC models. We did not perform an optimization for these models due to the time complexity of such models. However, a window of further research is possible using Faster DWT algorithms [76] similar to [77]. This will feasibly optimize these time series models.

## Figures and Tables

**Table 1:** The auxiliary features for image-CNN model.

Feature Name	Type	Feature Description
total_mouse_movements	Action-based	The total number of mouse movements.
total_scrolled_pixels _vertical		The total number of scrolled pixels.
total_clicks		The total number of mouse clicks.
total_keypresses		The total number of keyboard pressing.
total_pastes		The total number of pastes.
total_focus_changes		The total number of focusing changes.
total_pixels		The total number of pixels movements in x/y directions.
total_task_time	Time-based	The total time of completing the HIT.
total_on_foucs_time		The total time that was spent completing the HIT.
recorded_time_disparity		Difference between the total time and the time spent outside the HIT.
avg_dwell_time		Average time between two successive logged events.

**Table 2:** Accuracy and AUC-ROC for features vs. different importance thresholds.

Importance Threshold (Log10)	Mean/18 −11.156	Mean/9 −10.463	Mean/3 −9.365	Mean −8.266	Mean × 3 −7.1678	Mean × 9 −6.061	Mean × 18 −4.970
**No. of features**	1629	1158	984	770	377	78	41
**Accuracy**	82.9	83.3	83.1	82.1	82.8	80.6	78.2
**AUC-ROC**	81.1	81.3	80.9	82.0	81.1	80.8	79.1

**Table 3:** Hyper-parameter tuning.

Hyper-Parameter	Training Accuracy	Validation Accuracy	Training Loss	Validation Loss
Batch size	Learning rate: $10^{-4}$, Epochs: 150, Input Dimensions 32 × 32, dropout rate for conv. layer and dense layer = 0.5 and 0.25, respectively.			
25	0.8050	0.7633	0.4446	0.6291
**50**	0.7956	**0.7712**	0.4786	**0.5475**
75	0.7827	0.7539	0.4988	0.5517
Dropout rate (for the Conv. layer)	Batch size: 50, Learning rate: $10^{-4}$, Epochs: 150, Input Dimensions 32 × 32, dropout rate dense layer: 0.25.			
**0.25**	0.7485	**0.7649**	0.5225	**0.5264**
0.50	0.7587	0.7367	0.5306	0.5505
0.75	0.7254	0.7179	0.5523	0.5826
Dropout rate (for the Dense layer)	*Dropout rate: Conv. layer: 0.25, Batch size: 50, Learning rate:* $\;10^{-4}$ *, Epochs: 150, Input Dimensions 32 × 32*			
**0.25**	0.8054	**0.7821**	0.4551	**0.5423**
0.50	0.7391	0.7680	0.5495	0.5236
0.75	0.6865	0.6959	0.6121	0.6059
Image Input dimensions	Dropout rate: Conv. layer: 0.25, Batch size: 50, Learning rate:$\;10^{-4}$, Epochs: 150			
**32 × 32**	0.7705	**0.7837**	0.4987	**0.5080**
56 × 56	0.8195	0.7382	0.4271	0.5778
28 × 28	0.7991	0.7649	0.4695	0.5408

**Table 4:** Performance comparison against baselines.

Baseline\Performance Metric	Accuracy	AUC-ROC
DT_AF [32,34]	65.2	52.1
RF_AF [34]	66.6	53.0
DTW	65.0	50.0
TS-SVC	68.2	49.5
Feature-based model	**83.8**	**82.0**
Feature-based with reduction (avg. thresholds)	81.8	80.9
Image-CNN based model	76.6	72.3

## Data Availability

Not applicable.

## Appendix A

In this appendix, we present the details of the features that result from the feature selection stage of the feature-based model at Table A1.

**Table A1:** The features of the feature-based model.

Feature Name	Feature Description	Action Name	Importance Order	Parameters
Continuous Wavelet Transform coefficients	The Continuous Wavelet Transform for the Mexican hat wavelet	MMT *	1	1st Coefficient width 2
			8	1st Coefficient width 5
			15	1st Coefficient width 10
			18	1st Coefficient width 20
			63	2nd Coefficient width 5
			68	2nd Coefficient width 20
			71	2nd Coefficient width 10
			72	2nd Coefficient width 2
		FCT *	28	1st Coefficient width 10
			33	1st Coefficient width 2
			36	1st Coefficient width 20
			38	1st Coefficient width 5
		CST *	64	1st Coefficient width 2
			66	1st Coefficient width 10
			77	1st Coefficient width 5
Quantile	The q quantile of the sample (10 quantiles)	MMT	2	The 9th quantile
			6	The 1st quantile
			7	The 2nd quantile
			9	The 3rd quantile
			10	The 6th quantile
			11	The 7th quantile
			12	The 8th quantile
			14	The 4th quantile
			78	The 5th quantile
		FCT	22	The 1st quantile
			23	The 9th quantile
			25	The 8th quantile
			26	The 4th quantile
			27	The 6th quantile
			29	The 7th quantile
			32	The 3rd quantile
			34	The 2nd quantile
		CST	48	The 9th quantile
			52	The 8th quantile
			57	The 4th quantile
			59	The 2nd quantile
			73	The 6th quantile
			74	The 7th quantile
			75	The 1st quantile
Fast Fourier Transform coefficient	The fourier coefficients of the one-dimensional discrete Fourier Transform	MMT	16	Real 1st coefficient
			17	Absolute 1st coefficient
			76	Absolute 2nd coefficient
		FCT	30	Absolute 1st coefficient
			35	Real 1st coefficient
Sum values	The sum over the sample values	MMT	19	
		FCT	37	
Benford correlation	The correlation resulted from the Newcomb-Benford’s Law distribution	MMT	39	
		FCT	40	
		CST	56	
Absolute energy	The absolute energy of the sample which is the sum over the squared values	MMT	41	
Energy ratio by chunks	The sum of squares of chunk i out of N chunks expressed as a ratio with the sum of squares over the whole series. (10 segments)	MMT	43	First segment
			42	Second segment
Fast Fourier Transform aggregated	The spectral centroid (mean), variance, skew, and kurtosis of the absolute fourier transform spectrum.	MMT	44	Centroid
			61	Variance
Change quantiles	The average, absolute value of consecutive changes of the series x inside the corridor of quantiles q-low and q-high.	MMT	45	Mean without absolute difference of (the higher quantile and the lower quantile)
			55	Mean with absolute difference of (the higher quantile and the lower quantile)
Variation coefficient	The variation coefficient (standard error/mean, give relative value of variation around mean) of x	MMT	46	
Mean absolute change	The mean over the absolute differences between subsequent sample values	MMT	47	
Mean change	The mean over the differences between subsequent sample values.	MMT	49	
Linear trend	The linear least-squares regression for the values of the sample versus the sequence from 0 to length of the sample minus one.	MMT	50	Slope
			60	Intercept
Complexity Estimator	The estimation for a sample complexity (A more complex sample has more peaks, valleys etc.).	MMT	53	Without normalization
Standard deviation	The standard deviation of the sample x.	MMT	69	
Absolute sum of changes	The sum over the absolute value of consecutive changes in the series x.	MMT	62	
Maximum	The largest value of the sample x.	MMT	3	
		FCT	20	
		CST	70	
Minimum	The smallest value of the sample x.	MMT	4	
		FCT	21	
		CST	58	
Mean	The mean of the sample x	MMT	5	
		FCT	24	
		CST	54	
Median	The median of the sample x	MMT	13	
		FCT	31	
		CST	65	

* MMT: Mouse Movement Time, FCT: Focus Changes Time, CST: Clicks Specific Time.
